# Complex 45,X/46,XY Isochromosome Yp Mosaicism Presenting With Atypical Genitalia and an Incidental CYP21A2 Mutation: A Case Report

**DOI:** 10.7759/cureus.117061

**Published:** 2026-09-28

**Authors:** Fatima K Alqanea, Maryam M Almusalam, Rehab Amer, Lara Boustros, Minoosh Nasef

**Affiliations:** 1 Pediatric Endocrinology, King Hamad University Hospital, Muharraq, BHR; 2 Pediatrics, King Hamad University Hospital, Muharraq, BHR; 3 Neonatology, King Hamad University Hospital, Muharraq, BHR

**Keywords:** 45x/46xy mosaicism, ambiguous external genitalia, atypical genitalia, cyp21a2, disorder of sex development (dsd), hcg stimulation test, isochromosome yp, mixed gonadal dysgenesis

## Abstract

Differences of sex development (DSD) encompass a heterogeneous group of congenital conditions characterized by atypical development of chromosomal, gonadal, or anatomical sex. Mosaicism involving 45,X/46,XY cell lines is a rare cytogenetic variant that presents with a broad phenotypic spectrum ranging from complete male or female phenotypes to mixed gonadal dysgenesis and atypical genitalia. We report a neonate, the product of a twin pregnancy, born at 36 weeks of gestation who presented at birth with atypical genitalia, including a phallus with ventral chordee, penoscrotal hypospadias, and an asymmetric, nonpalpable right gonad. Initial imaging demonstrated bilateral intra-abdominal/inguinal gonads alongside persistent, hypoplastic Müllerian structures (uterus, cervix, and upper vagina). Cytogenetic evaluation revealed a complex four-cell-line mosaicism: 46,XY, monosomy X, isochromosome Yp, and 47,XY,i(Y)(p10). Whole-exome sequencing confirmed the DSD diagnosis and additionally identified a coincidental heterozygous carrier status for two pathogenic alleles localized in cis with a normal CYP21A2 gene copy. Biochemical evaluation, including 17-hydroxyprogesterone, adrenocorticotropic hormone, cortisol, and serum electrolytes, showed no evidence of active congenital adrenal hyperplasia. Human chorionic gonadotropin stimulation testing demonstrated a robust testosterone response, supporting preserved Leydig cell function. Following a comprehensive multidisciplinary assessment and counseling with the family, an individualized plan for sex of rearing and long-term clinical follow-up was established. This case highlights the importance of distinguishing disease-causing enzymatic defects from incidental carrier states when evaluating complex DSD. Integrating cytogenetic findings with biochemical and functional endocrine assessment can help clarify the underlying diagnosis and gonadal function, thereby providing individualized multidisciplinary care, including appropriate surveillance and consideration of future surgical and gonadal management.

## Introduction

Differences of sex development (DSD) represent a complex spectrum of congenital conditions wherein chromosomal, gonadal, or phenotypic sex development is atypical. The overall incidence of DSD is estimated at one in 1,000 to one in 4,500 live births [1]. Among chromosomal DSDs, 45,X/46,XY mosaicism is a recognized etiology associated with mixed gonadal dysgenesis. Phenotypic manifestations depend heavily on the tissue distribution and relative predominance of the 45,X versus Y-bearing cell lines during embryonic morphogenesis [2,3].

The presentation of atypical genitalia at birth routinely necessitates rapid diagnostic evaluation to exclude life-threatening conditions, most notably classical salt-wasting congenital adrenal hyperplasia (CAH) secondary to 21-hydroxylase deficiency (CYP21A2 mutations) [4,5]. Coexisting abnormalities affecting sex-chromosome constitution and adrenal steroidogenesis have rarely been described in patients with DSD. Accordingly, the identification of a CYP21A2 variant in an individual with a chromosomal DSD should be interpreted in the context of zygosity, biochemical findings, and the overall phenotype to determine whether it represents a disease-causing abnormality or an incidental carrier finding [6].

Herein, we report a complex case of a neonate presenting with atypical genitalia who was found to have four-cell-line 45,X/46,XY mosaicism involving an isochromosome Yp (i(Y)(p10)), alongside a heterozygous CYP21A2 variant considered an incidental carrier finding. The patient was clinically stable and did not show laboratory or clinical instability suggestive of active CAH. This case highlights the importance of integrating cytogenetic, molecular, and endocrine findings within a multidisciplinary framework to establish the underlying diagnosis and guide individualized clinical management and long-term follow-up.

## Case presentation

A neonate at 36 weeks and two days of gestation, born as Twin A via elective cesarean section due to breech presentation and maternal premature rupture of membranes, was admitted to the neonatal intensive care unit (NICU) for evaluation of atypical genitalia. The co-twin (Twin B) was a healthy dichorionic, diamniotic female twin with normal female external genitalia. The mother was a 28-year-old gravida 3, para 2 woman with an unremarkable past medical and family history. Delivery was uncomplicated, and the neonate demonstrated normal Apgar scores with a birth weight of 2.215 kg.

On initial physical examination, the neonate was clinically and vitally stable without dysmorphic features. Genital examination revealed a phallus measuring 1.5 cm in length, with complete measurement restricted by ventral chordee. There was no hyperpigmentation of the genital skin, and no external vaginal orifice was observed. The labioscrotal folds demonstrated a bifid scrotum with a single perineal urethral meatus, consistent with penoscrotal hypospadias. Palpation revealed a discrete gonad in the left inguinal fold, whereas the right gonad was nonpalpable (Figure 1).

**Figure 1:**
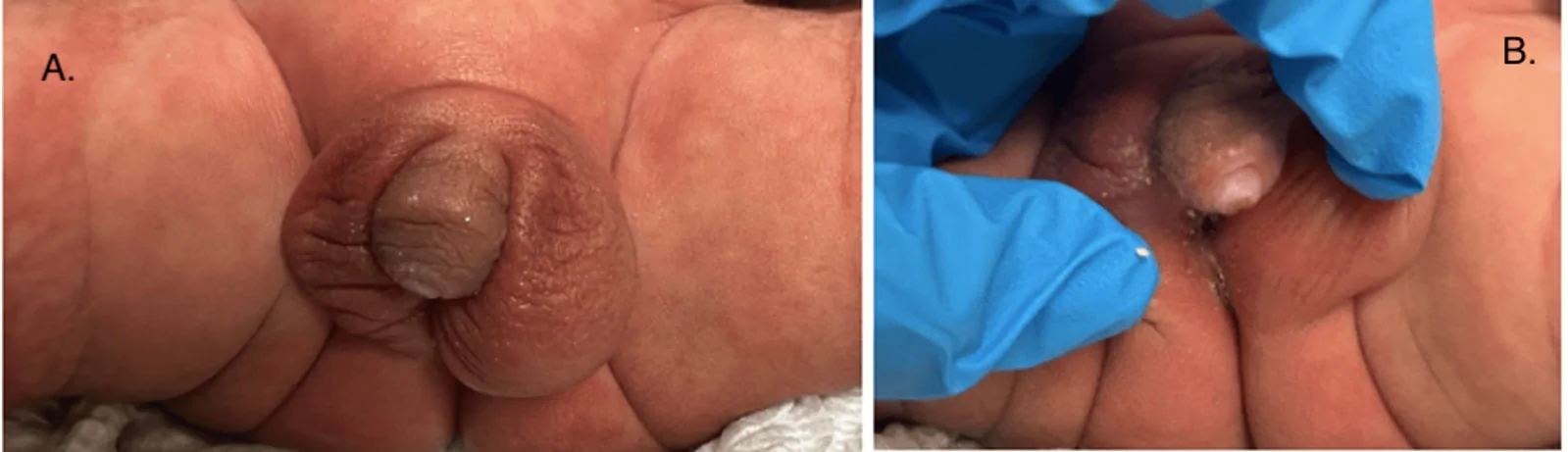
External genital findings on neonatal examination. (A) Anterior view demonstrating an atypical genital appearance, with asymmetric labioscrotal folds and a palpable left-sided gonad. (B) Ventral view demonstrating penoscrotal hypospadias, with the external urethral opening located at the penoscrotal junction.

Given the clinical presentation of atypical genitalia, the patient was immediately monitored to exclude classical salt-wasting CAH. Serial blood pressure measurements, serum electrolytes, and blood glucose levels remained within normal parameters throughout the NICU stay. Arterial blood gas analyses consistently demonstrated no metabolic acidosis. The neonate fed well orally and passed both urine and meconium normally.

Peripheral blood karyotyping revealed a complex four-cell-line mosaicism: 45,X; 46,XY; 46,X,i(Y)(p10); and 47,XY,i(Y)(p10). Monosomy X (45,X) constituted the predominant cell population (73%), while 27% of analyzed cells contained Y-chromosome material, including standard 46,XY, an isochromosome of the short arm of the Y chromosome (i(Y)(p10)), and a cell line with an extra Y chromosome. Fluorescence in situ hybridization (FISH) confirmed the presence of the sex-determining region Y (SRY) gene on the short arm of the Y chromosome, which could promote testicular development. However, the degree of virilization correlates with the distribution of Y-containing cells in the tissues. The karyotype findings suggest a complex DSD, classified as mixed gonadal dysgenesis with atypical genitalia (Figure 2).

**Figure 2:**
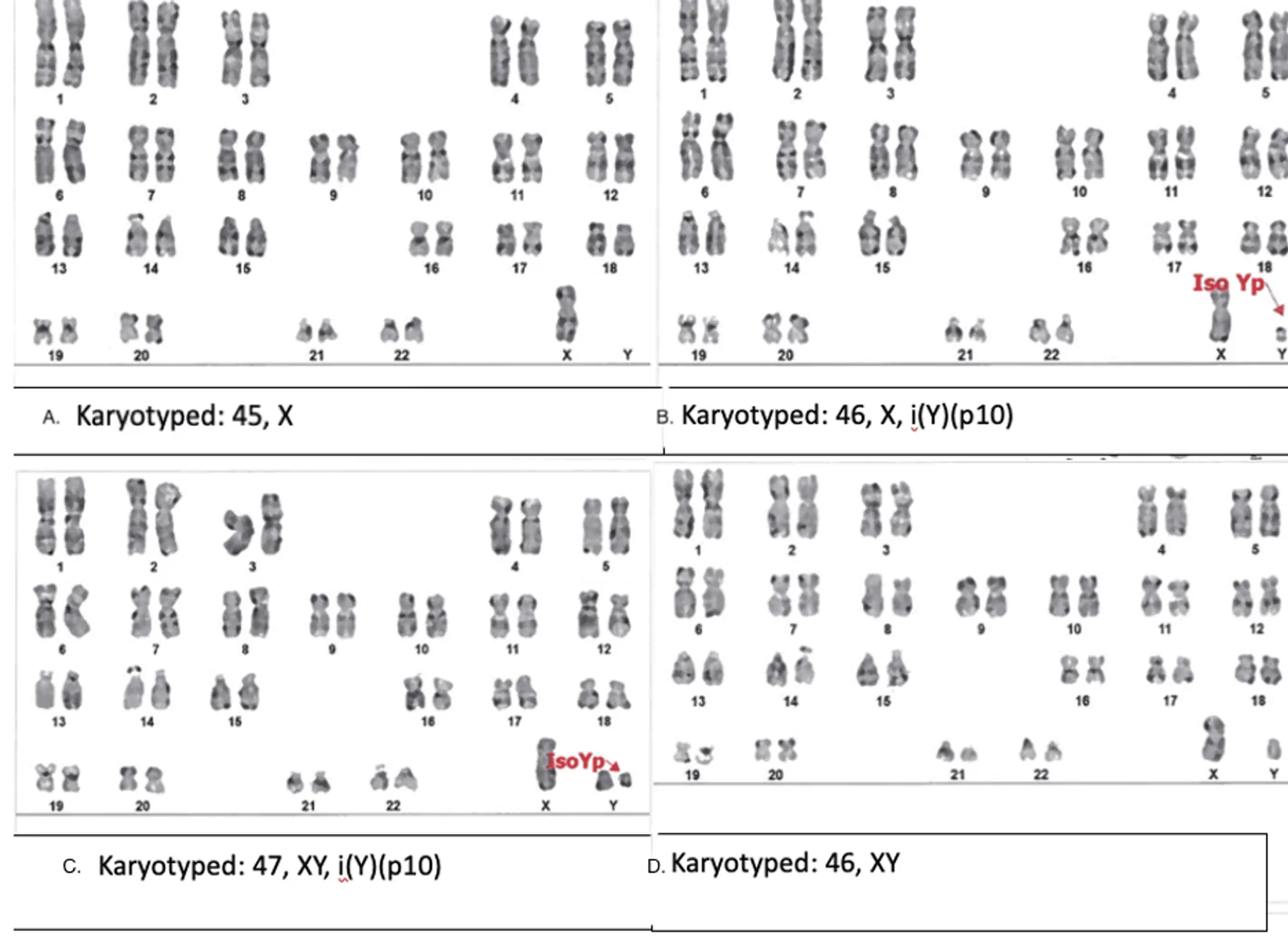
Representative karyograms demonstrating four-cell-line sex-chromosome mosaicism. Representative G-banded karyograms from peripheral blood lymphocytes showing four distinct cell lines: (A) 45,X; (B) 46,X,i(Y)(p10), containing an isochromosome of the short arm of the Y chromosome; (C) 47,XY,i(Y)(p10), containing both a normal Y chromosome and an additional i(Y)(p10); and (D) 46,XY. The red arrows indicate the Y-derived isochromosome. i(Y)(p10) denotes an isochromosome composed of two copies of the short arm of the Y chromosome with a centromeric breakpoint at p10. Collectively, these findings confirm four-cell-line sex-chromosome mosaicism.

Pelvic and scrotal ultrasonography identified a left gonad measuring 4 × 6 × 8 mm located within the left inguinal canal and a contralateral right gonad of identical dimensions (4 × 6 × 8 mm) situated intra-abdominally, adjacent to the right external iliac artery. A right-sided inguinal hernia containing mobile bowel loops was also identified. Dedicated screening via echocardiography and renal ultrasonography was performed to evaluate for 45,X-associated anomalies, revealing a normal aortic arch, trileaflet aortic valve, and structurally normal bilateral kidneys. Additionally, pelvic ultrasound imaging detected a midline, oblong, tubular structure posterior to the bladder measuring 3.0 × 0.4 cm, suspicious for Müllerian structures (hypoplastic uterus/cervix). No definite ovarian tissue was identified (Figure 3).

**Figure 3:**
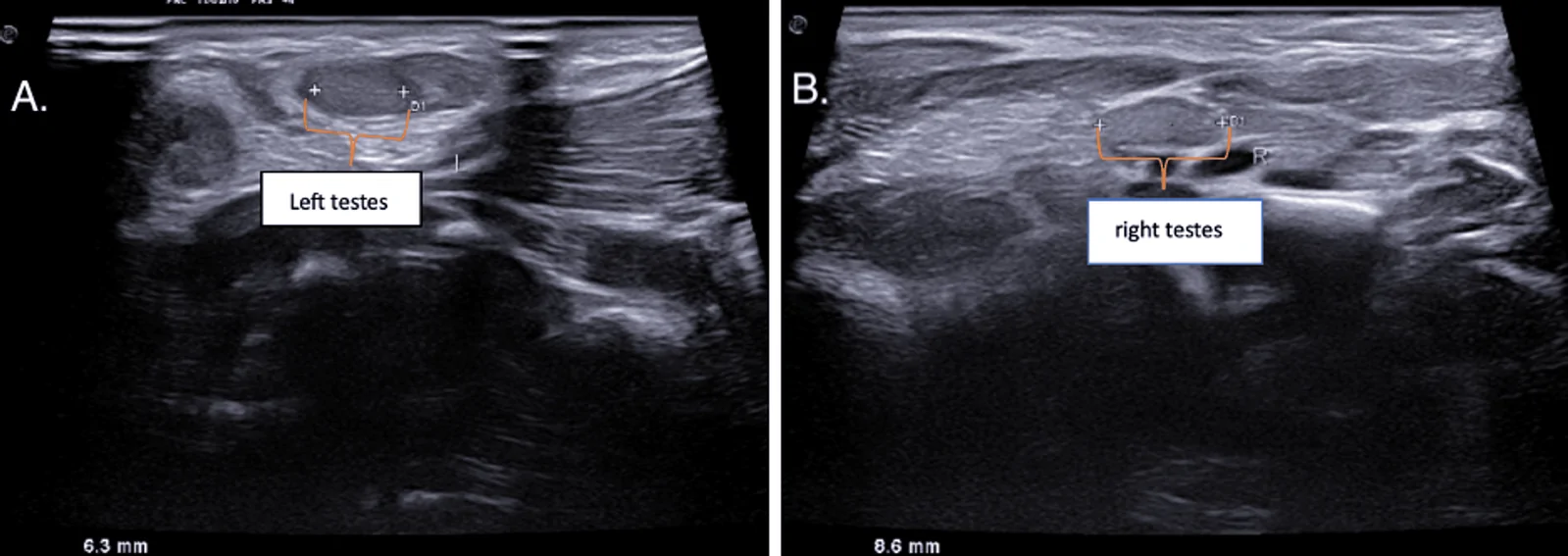
Ultrasonographic identification of bilateral gonads. Grayscale ultrasonographic images demonstrating (A) the left gonad within the left inguinal canal and (B) the right gonad intra-abdominally. Each gonad measured approximately 4 × 6 × 8 mm and demonstrated sonographic features consistent with testicular tissue. The orange calipers delineate the measured borders of each gonad.

A complementary pelvic MRI scan confirmed the presence of a hypoplastic, tubular uterine structure with a thin endometrial lining, a narrow cervix, and a distal, blind-ending, fluid-filled vaginal vault. Neither testicles nor ovaries could be definitively characterized on MRI due to nonspecific tissue signal, underscoring the necessity of cytogenetic and endocrine profiling (Figure 4).

**Figure 4:**
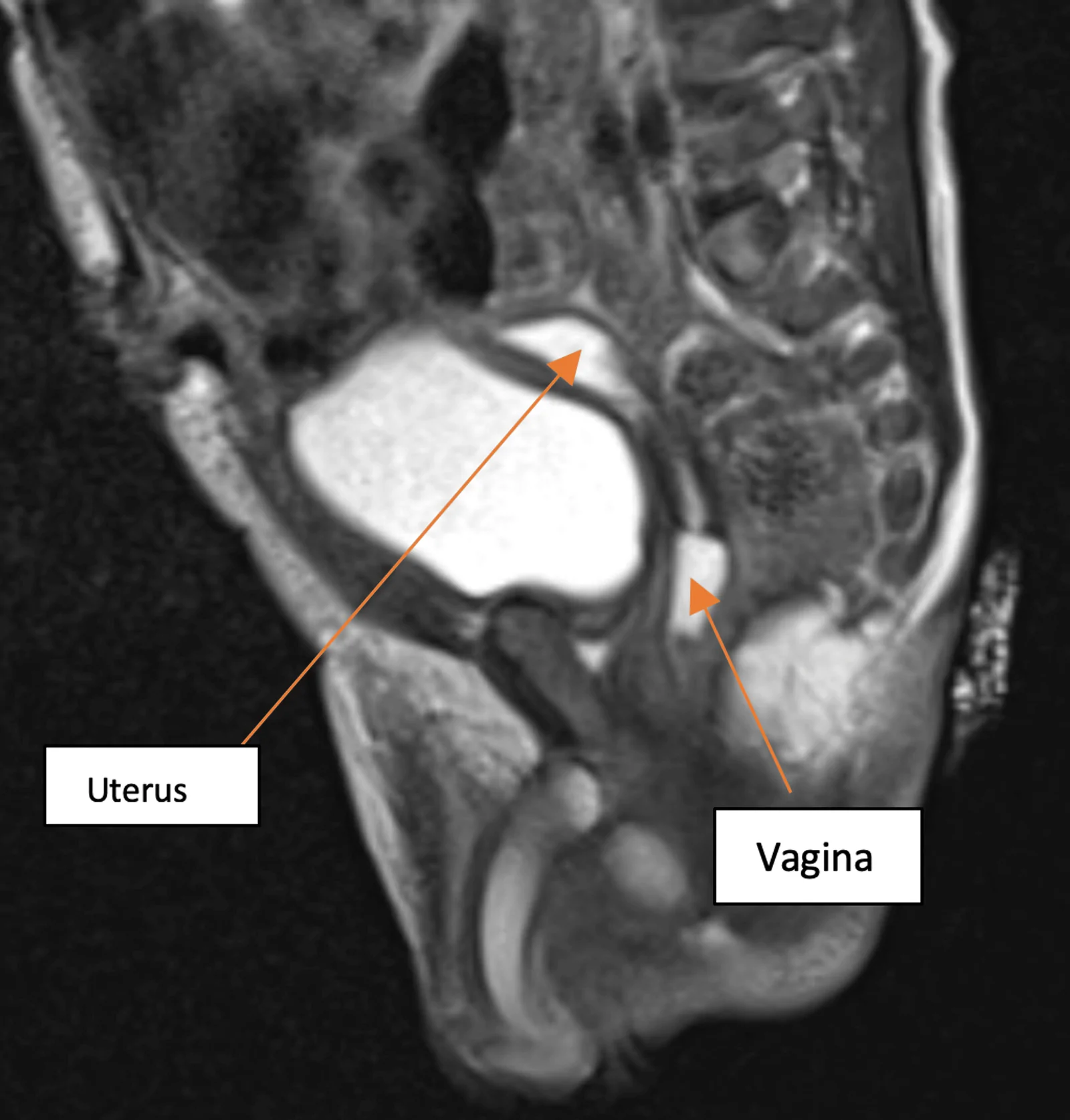
Pelvic MRI demonstrating Müllerian structures. Mid-sagittal T2-weighted MRI of the pelvis demonstrating the presence of a uterus and vagina. The orange arrows identify the uterus, located posterior to the urinary bladder, and the vagina extending inferiorly toward the perineum.

Prior to obtaining the karyotype and proceeding with imaging, a baseline hormonal and endocrine panel was collected on the patient’s second day of life. This was done to rule out major endocrine conditions associated with atypical genitalia, and the results are shown in Table 1. Serum adrenocorticotropic hormone (ACTH) and cortisol levels were normal. The serum 17-hydroxyprogesterone (17-OHP) level was also measured. The androgenic hormone panel revealed a testosterone (T) level of 4.72 nmol/L (normal range, 2.6-13.9 nmol/L) and a dihydrotestosterone (DHT) level of 32.2 ng/dL (normal range, <120 ng/dL). The T/DHT ratio was calculated as 3.76, which is relatively low, making 5α-reductase deficiency unlikely. The anti-Müllerian hormone level was 27 ng/mL (reference range, 18-283 ng/mL), consistent with preserved Sertoli cell function.

**Table 1:** Relevant hormonal and enzymatic investigations collected on days 2 and 27 of life. The isolated elevation of 17-OHP could be explained by the use of a chemiluminescent immunoassay, which can overestimate 17-OHP in newborns because of steroid cross-reactivity. Other influential factors include stress and illness. Elevation should prompt CYP21A2 gene testing. ACTH: adrenocorticotropic hormone; AMH: anti-Müllerian hormone; DHEA-S: dehydroepiandrosterone sulfate; FSH: follicle-stimulating hormone; LH: luteinizing hormone; 17-OHP: 17-hydroxyprogesterone

Investigation	Second day of life	27th day of life	Hospital reference range	Interpretation
ACTH	12.4 pg/mL	x	7.2-63.3 pg/mL	Normal
Cortisol	8.4 µg/dL	x	6.99-25 µg/dL	Normal
17-OHP	14.2 ng/mL	x	<6.3 ng/mL	Isolated elevation
Androstenedione	0.48 ng/mL	0.98 ng/mL	0.2-2.9 ng/mL	Normal
DHEA-S	14.71 µmol/mL	10.5 µmol/mL	<16.48 µmol/mL	Normal
Dihydrotestosterone	32.2 ng/dL	x	<120 ng/dL	Normal
Testosterone	4.72 nmol/L	Before stimulation: 4.29 nmol/L; after stimulation: 7.74 nmol/L	0.09-3.35 nmol/L	Increased 1.8-fold from baseline
AMH	-	27 ng/mL	18-283 ng/mL	Normal
FSH	-	6.36 mIU/mL	1.4-18 mIU/mL	Normal
LH	-	4.48 mIU/mL	1.5-9.3 mIU/mL	Normal

On day 27 of life, the patient underwent a human chorionic gonadotropin (hCG) stimulation test to evaluate the presence and functional capacity of Leydig cells to produce testosterone. Baseline hormone levels demonstrated an elevated luteinizing hormone level of 4.48 mIU/mL and a follicle-stimulating hormone level of 6.36 mIU/mL, reflecting physiological mini-puberty of infancy. Baseline testosterone was 4.29 nmol/L, and the remainder of the investigations was unremarkable.

The patient received intramuscular hCG (500 IU) once daily for three consecutive days. Twenty-four hours following the final dose, which was on day 4 post-stimulation, serum testosterone rose significantly to 7.74 nmol/L, confirming responsive, functional Leydig cell tissue capable of androgen biosynthesis, as shown in Table 1.

In view of the chromosomal mosaicism, whole-exome sequencing (WES) was sent earlier as part of the initial workup to further evaluate whether any genetic disorder was associated with it. The results were released approximately 6 weeks later and confirmed the presence of 45,X/46,XY DSD, along with a coincidental heterozygous carrier state for two pathogenic CYP21A2 variants localized in cis with a normal CYP21A2 gene copy encoding 21-hydroxylase.

Following completion of the diagnostic evaluation, the case was reviewed by a multidisciplinary team comprising pediatric endocrinology, pediatric surgery, neonatology, pediatric radiology, medical genetics, and clinical ethics, in discussion with the family. The overall clinical, anatomical, cytogenetic, and endocrine findings, including evidence of functional testicular tissue and a robust testosterone response to hCG stimulation, were considered collectively, and male sex of rearing was selected and agreed upon by the parents. The patient was subsequently discharged in stable condition with planned pediatric surgical follow-up for management of hypospadias, ventral chordee, and undescended testes, together with ongoing multidisciplinary follow-up and surveillance of gonadal health.

## Discussion

The presence of 45,X/46,XY mosaicism presents a wide spectrum of clinical phenotypes, ranging from phenotypic females with Turner syndrome features to individuals with atypical genitalia, mixed gonadal dysgenesis, or fully virilized males [2,3]. In our patient, the predominant 45,X cell line (73%) accounted for the incomplete virilization and persistence of Müllerian structures (hypoplastic uterus and upper vagina), while the 27% Y-chromosome-bearing cell lines supplied sufficient SRY expression to initiate testicular differentiation and partial androgen production. Gonadal differentiation mainly depends on SRY gene expression. In males, its expression drives testicular development, whereas in females, its absence allows ovarian development [7].

The structural anomaly observed in the Y chromosome, an isochromosome of the short arm (i(Y)(p10)), results in duplication of Yp (containing SRY) and loss of Yq. Loss of Yq regions containing the azoospermia factor loci predictably impacts future spermatogenesis, a key counseling point for the family regarding long-term fertility [8]. A critical element of this case was distinguishing the incidental genetic finding from the functional etiology of the atypical genitalia. WES identified a heterozygous carrier state for two CYP21A2 gene variants. Because CAH is a primary cause of atypical genitalia in neonates, initial clinical focus naturally centered on steroidogenesis defects. However, serial metabolic monitoring alongside the available 17-OHP, ACTH, cortisol, and electrolyte findings supported the absence of active CAH, establishing that the CYP21A2 finding was an incidental carrier state rather than the primary cause of the phenotypic presentation. Despite the fact that the literature does not point to a clear association between CAH and genetic mosaicism, their coexistence has been noted in several published cases [9,10]. Within the published cases of DSD with CAH gene mutations, most cases presented with clinical and laboratory findings suggestive of CAH. However, in our patient, the finding was incidental and identified through genetic testing.

The hCG stimulation test was pivotal in clinical decision-making [11]. The post-stimulation increase in testosterone (from 4.29 nmol/L to 7.74 nmol/L) confirmed the presence of functional Leydig cells capable of responding to gonadotropin stimulation. A robust testosterone response after stimulation has been demonstrated in our patient. This is concluded if testosterone levels either increase to double the baseline level or increase above the upper limit of the prepubertal range [11]. This endocrine response confirms the functionality of the testicular tissue, combined with phallic structure and predominantly male internal anatomical features.

Long-term management for patients with dysgenetic gonads and Y-chromosome-containing cell lines requires structured surveillance due to the increased risk of malignant transformation, particularly gonadoblastoma and dysgerminoma [12]. Surgical planning includes orchidopexy for intra-abdominal or inguinal gonads to enable serial clinical and ultrasonographic examination, staged repair of penoscrotal hypospadias and chordee, and ongoing psychological support to address gender identity and developmental milestones. The patient is planned for laparoscopic exploration and bilateral gonadal tissue biopsy with pediatric surgery, together with long-term pediatric endocrine follow-up. Psychological support is crucial in patients who are born with atypical genitalia, and follow-up should be tailored according to the underlying cause. Many published papers, including literature reviews, have noted a degree of gender dysphoria among patients with CAH. One paper included 19 cases of patients with CAH who were assigned female at birth and had socially transitioned to male [13]. Similarly, this has been observed in cases of 5α-reductase deficiency [14]. In DSD cases, gender dysphoria has also been reported in some patients and was slightly more common among females than males [15,16].

## Conclusions

This case demonstrates the complex diagnostic evaluation required for neonates presenting with atypical genitalia secondary to 45,X/46,XY isochromosome Yp mosaicism. It underscores the importance of correlating whole-exome findings with clinical and endocrine data to avoid misattributing phenotypes to nonpathogenic carrier states, such as heterozygous CYP21A2 mutations. Functional testicular assessment via hCG stimulation testing, alongside cytogenetic profiling, provides essential objective parameters to guide multidisciplinary decisions regarding sex of rearing, surgical reconstruction, and protocolized long-term oncological surveillance.
